# The association between body mass index and postpartum hemorrhage after cesarean delivery

**DOI:** 10.1038/s41598-023-38526-7

**Published:** 2023-07-25

**Authors:** Julia Whitley, Wayde Dazelle, Shawn Kripalani, Homa Ahmadzia

**Affiliations:** Department of Obstetrics and Gynecology, George Washington University School of Medicine and Health Sciences, 2150 Pennsylvania Ave NW, Washington, DC 20037 USA

**Keywords:** Epidemiology, Outcomes research, Public health

## Abstract

We aimed to evaluate the association between obesity and postpartum hemorrhage (PPH) after cesarean delivery (CD). This was a retrospective cohort study using a multicenter database of 20 hospitals in the United States. We analyzed 27,708 patients undergoing CD from 2015 to 2019. The exposure of interest was BMI, and the primary outcome was PPH (estimated blood loss [EBL] ≥ 1000 mL). Simple logistic regression was used to evaluate the relationship between obesity and intrapartum complications. Multivariable logistic regression was used to adjust for any confounding demographic variables. Hosmer and Lemeshow’s purposeful selection algorithm was adapted to develop a multivariable logistic regression model of PPH. Analyses were conducted using STATA 16.1 (College Station, Texas) with *p* ≤ 0.05 considered significant. BMI exerted a significant effect on the frequency of PPH (*p* = 0.004). Compared to patients with BMI 18.5–24.9 kg/m^2^, patients with BMI between 25 and 59.9 kg/m^2^ had an increased odds of PPH. The odds of PPH in patients with BMI > 60 kg/m^2^ was not increased compared to patients with BMI 18.5–24.9 kg/m^2^. Obesity was associated with a decreased odds of blood transfusion (aOR 0.73, 95% CI 0.55–0.97). In conclusion, higher BMI was associated with PPH yet a lower odds of transfusion after CD.

## Introduction

Obesity (defined as body mass index [BMI] ≥ 30 kg/m^2^) is one of the most common pregnancy complications, affecting more than one-third of women of reproductive age^1,2^. In addition, the prevalence of obesity and severe obesity (BMI of 40 kg/m^2^ or greater) is increasing in the United States^2^. Obesity has been associated with multiple adverse pregnancy outcomes, including pregnancy loss, gestational diabetes, venous thromboembolism, and increased risk for cesarean delivery (CD)^3–6^.

Postpartum hemorrhage (PPH) is the leading cause of maternal mortality worldwide, accounting for more than 80 thousand maternal deaths in 2015^7^. Obesity has been considered to be a potential risk factor for PPH for physiologic reasons; animal models have demonstrated a decreased ability to maintain blood pressure in response to hemorrhage among rats with obesity^8^. Obesity is also associated with other factors that increase the risk of blood loss, including CD and surgical tissue injury^3,9^. However, others have hypothesized that the pro-inflammatory and relative hypercoagulable state found in patients with obesity may actually be protective against increased blood loss^10^. While obesity is often cited as a risk factor for uterine atony and PPH, the obstetric literature is mixed in regard to the effect of BMI on PPH^11,12^. In a large cohort in Sweden, the risk of atonic PPH increased with increasing BMI regardless of mode of delivery, with a twofold increased risk of PPH in patients with class III obesity^13^. In contrast, one retrospective cohort study in California reported a decreased odds of severe hemorrhage after CD^9^. Furthermore, in a recent systematic review and meta-analysis of studies including patients who developed atonic PPH, obesity was not found to be associated with atonic PPH^14^.

The primary objective of our study was to determine if obesity is associated with an increased odds of PPH or hemorrhage-related complications (including blood transfusion) during delivery. To eliminate the potential confounding effect of mode of delivery and its associated variables on PPH, only women undergoing CD were eligible for inclusion in this study. To our knowledge, this is the first multicenter retrospective cohort study specifically examining the effect of obesity on PPH after CD.

## Materials and methods

Data were obtained from a multicenter database of women admitted to labor and delivery units from 2015 to 2019 in the Universal Health Services (UHS) system in the United States. All patients who underwent CD during their admission were eligible for inclusion in this study (N = 38,334). A total of 8252 patients were excluded from analysis due to missing data for BMI at time of delivery as this was the exposure of interest. Of those excluded for missing BMI at delivery, approximately 65% were missing data for ≥ 60% of analysis-relevant variables. An additional 2374 patients in the dataset with vacuum- or forceps-assisted delivery were also excluded to ensure that patients who had a vaginal delivery were not included in the analysis. A total of 27,708 patients were eligible for inclusion in the analysis. CD were reported by 20 hospitals across the Eastern (N = 5; 18.2%), Western (N = 7; 48%), and Central (N = 8; 33.8%) United States. The requirement for informed consent was waived by The George Washington University Committee on Human Research Institutional Review Board due to the retrospective nature of the study, and all data were de-identified prior to analysis. The study was determined to be research that is exempt from IRB review by The George Washington University Committee on Human Research Institutional Review Board under Department of Health and Human Services (DHHS) regulatory category 4 (IRB number NCR203136). All methods were carried out in accordance with relevant guidelines and regulations. No experimental protocols were performed as this was a retrospective study.

The main exposure of interest was BMI at time of delivery. BMI at delivery was sorted into six categories (BMI below 18.5, 18.5 to 24.9, 25.0 to 29.9, 30.0 to 34.9, 35.0 to 39.9, and above 40) according to guidelines from the National Institutes of Health and World Health Organization^1,15^, of which 5 were employed for descriptive and demographic analysis (Table 1). In the primary analysis, maternal BMI was treated as a dichotomous categorical variable to evaluate obesity (BMI ≥ 30 kg/m^2^). The primary outcome was PPH, defined as EBL ≥ 1000 mL for the purpose of analysis. Additional outcomes such as estimated gestational age (EGA) less than 37 weeks, intrapartum complications including pre-eclampsia and chorioamnionitis, abnormal hematologic parameters including thrombocytopenia, and pharmacologic interventions including oxytocin use were evaluated as secondary outcomes. Descriptive statistics included counts, proportions, means, and standard deviations calculated as necessary using standard definitions. To evaluate the association between BMI classification and other patient-level variables, we used chi-square or Fischer’s exact test (when N ≤ 5) for categorical variables and analysis of variance for continuous variables^16^.

**Table 1:** Demographic factors of study cohort.

Maternal characteristics	All (n = 27,708)	BMI 18.5–24.9 (n = 2101)	BMI 25–29.9 (n = 7407)	BMI 30–34.9 (n = 8406)	BMI 35–39.9 (n = 5351)	BMI > 40 (n = 4443)	*p* value
Age (y), Mean ± SD	28.9 ± 5.95						
< 20	1278 (4.6%)	153 (7.3%)	366 (4.9%)	386 (4.6%)	230 (4.3%)	143 (3.2%)	** < 0.001**
20–34	20,912 (75.5%)	1586 (75.5%)	5484 (74.0%)	6307 (75.0%)	4085 (76.3%)	3450 (77.7%)	
> 35	5291 (19.1%)	343 (16.3%)	1498 (20.2%)	1649 (19.6%)	985 (18.4%)	816 (18.4%)	
Unknown	227 (0.8%)	19 (0.9%)	59 (0.8%)	64 (0.8%)	51 (1.0%)	34 (0.8%)	
BMI, Median (Range)	32.4 (18.6–70.0)						
Race/Ethnicity							
Black	3578 (12.9%)	276 (13.1%)	828 (11.2%)	1015 (12.1%)	738 (13.8%)	721 (16.2%)	** < 0.001**
White	15,248 (55.0%)	1124 (53.5%)	4121 (55.6%)	4575 (54.4%)	2986 (55.8%)	2442 (55.0%)	
Asian	1174 (4.2%)	155 (7.4%)	507 (6.8%)	318 (3.8%)	137 (2.6%)	57 (1.3%)	
Other/Unknown	7708 (27.8%)	546 (26.0%)	1951 (26.3%)	2498 (29.7%)	1490 (27.8%)	1223 (27.5%)	
Marital status							
Single	12,529 (45.2%)	979 (46.6%)	3135 (42.3%)	3719 (44.2%)	2466 (46.1%)	2230 (50.2%)	** < 0.001**
Married	13,598 (49.1%)	989 (47.1%)	3863 (52.2%)	4189 (49.8%)	2600 (48.6%)	1957 (44.1%)	
Other/Unknown	1581 (5.7%)	133 (6.3%)	409 (5.5%)	498 (5.9%)	285 (5.3%)	256 (5.8%)	
Geographical area							
East Coast (n = 5)	5044 (18.2%)	373 (17.8%)	1378 (18.6%)	1512 (18.0%)	955 (17.9%)	826 (18.6%)	** < 0.001**
Central (n = 8)	9377 (33.8%)	679 (32.3%)	2331 (31.5%)	2862 (34.1%)	1901 (35.5%)	1604 (36.1%)	
West coast (n = 7)	13,287 (48.0%)	1049 (49.9%)	3698 (49.9%)	4032 (48.0%)	2495 (46.6%)	2013 (45.3%)	
Insurance							
Medicaid	7503 (27.1%)	542 (25.8%)	1838 (24.8%)	2210 (26.3%)	1575 (29.4%)	1338 (30.1%)	** < 0.001**
Private	11,754 (42.4%)	898 (42.7%)	3287 (44.4%)	3604 (42.9%)	2181 (40.8%)	1784 (40.2%)	
Uninsured	988 (3.6%)	111 (5.3%)	335 (4.5%)	317 (3.8%)	144 (2.7%)	81 (1.8%)	
Other/Unknown	7463 (26.9%)	550 (26.2%)	1947 (26.3%)	2275 (27.1%)	1451 (27.1%)	1249 (27.9%)	
Parity							
0	5035 (18.2%)	469 (22.3%)	1385 (18.7%)	1518 (18.1%)	910 (17.0%)	753 (17.0%)	** < 0.001**
1	13,981 (50.5%)	1091 (51.9%)	3959 (53.5%)	4216 (50.2%)	2605 (48.7%)	2110 (47.5%)	
2	4018 (14.5%)	267 (12.7%)	975 (13.2%)	1213 (14.4%)	831 (15.5%)	732 (16.5%)	
3	4070 (14.7%)	232 (11.0%)	944 (12.7%)	1287 (15.3%)	869 (16.2%)	738 (16.6%)	
Missing/Unknown	604 (2.2%)	42 (2.0%)	144 (1.9%)	172 (2.1%)	136 (2.5%)	110 (2.5%)	
Number of prior CD							
0	22,148 (79.9%)	1690 (80.4%)	6007 (81.1%)	6742 (80.2%)	4206 (78.6%)	3503 (78.8%)	0.121
1	3137 (11.3%)	249 (11.9%)	802 (10.8%)	943 (11.2%)	620 (11.6%)	523 (11.8%)	
2	1641 (5.9%)	119 (5.7%)	412 (5.6%)	485 (5.8%)	349 (6.5%)	276 (6.2%)	
3+	782 (2.8%)	43 (2.0%)	186 (2.5%)	236 (2.8%)	176 (3.3%)	141 (3.2%)	
Asthma	470 (1.7%)	30 (1.4%)	80 (1.1%)	109 (1.3%)	114 (2.1%)	137 (3.1%)	** < 0.001**
Pre-gestational diabetes	567 (2.1%)	16 (0.8%)	76 (1.0%)	147 (1.8%)	148 (2.8%)	180 (4.1%)	** < 0.001**
Chronic hypertension	670 (2.4%)	11 (0.5%)	73 (1.0%)	132 (1.6%)	195 (3.6%)	259 (5.8%)	** < 0.001**
Cardiac disease	3 (0.01%)	0 (0.0%)	0 (0.0%)	1 (0.01%)	0 (0.0%)	2 (0.05%)	0.231
Renal disease	16 (0.06%)	0 (0.0%)	3 (0.04%)	5 (0.06%)	4 (0.07%)	4 (0.09%)	0.673
Tobacco use	4082 (14.7%)	343 (16.3%)	1074 (14.5%)	1242 (14.8%)	731 (13.7%)	692 (15.6%)	**0.018**
History of PPH	470 (1.7%)	37 (1.8%)	120 (1.6%)	150 (1.8%)	87 (1.6%)	76 (1.7%)	0.929

*Y* year, *SD* standard deviation, *BMI* body mass index, *CD* cesarean delivery, *PPH* postpartum hemorrhage.

Significant values are in bold.

We employed simple logistic regression to evaluate the relationship between obesity and intrapartum complications, including PPH. We used multivariable logistic regression to adjust for any confounding demographic characteristics that were associated with obesity at the 0.1 alpha level. To determine the demographic and intrapartum variables most associated with PPH, we adapted Hosmer and Lemeshow’s purposeful selection algorithm to develop a multivariable logistic regression model of PPH^17,18^. Candidate variables were screened with simple logistic regression, using the Wald test and a p-value cutoff of ≤ 0.25 to exclude variables from consideration. During the iterative process of variable selection, covariates were sequentially removed from the model if they were not significant at the 0.1 alpha level and did not exert a confounding influence. We tested for multicollinearity between covariates by determining the variance inflation factor. A literature review was also used to support the choice of covariates as potential confounders influencing the odds of PPH^19–23^.

EBL was not reported for 1918 deliveries (6.92%), maternal age was not reported for 227 deliveries (0.82%), and EGA was not reported for 451 deliveries (1.63%), with approximately 9% of deliveries missing any combination of these variables. Because these data could not be assumed to be missing completely at random, we treated them as missing at random and employed multiple imputation by chained equations with 10 total imputations to account for statistical uncertainty while addressing missing data^24^. Missing data were also observed for race/ethnicity, marital status, insurance type, and parity. Missing data from these variables were included in the multivariable models as dummy variables. All analyses were conducted using STATA 16.1 (College Station, Texas) with *p* ≤ 0.05 considered significant.

## Results

Our cohort included 27,708 CD from 20 hospitals in the Universal Health Services (UHS) system across the United States. The majority of patients included in our study were White (55.0%) and between ages 20 and 34 years (75.5%); 49.1 percent were married. A large proportion of the patients in our cohort had private insurance (42.4%) and delivered at hospitals in the West Coast (48.0%). The majority of patients (79.9%) had no prior CD. We compared demographic and medical characteristics across 5 BMI categories, with several differences noted between groups. The prevalence of asthma, pre-gestational diabetes, and chronic hypertension increased as BMI increased between groups (*p* < 0.001 for all) (Table 1).

Concerning our primary outcome of interest, obesity in its dichotomized form (defined as BMI ≥ 30 kg/m^2^) did not significantly influence the odds of EBL ≥ 1000 mL. Increasing BMI category was, however, associated with EBL ≥ 1000 mL (*p* < 0.001) (Table 2). While categorical obesity did not influence the odds of EBL ≥ 1000 mL, we conducted a sensitivity analysis to gauge the extent to which the extremes of BMI, relative to BMI 18.5–24.9 kg/m^2^, influence the odds of EBL ≥ 1000 mL in this population. The odds of EBL ≥ 1000 mL increased with BMI between 25 and 59.9 kg/m2 relative to that observed in patients with BMI 18.5–24.9 kg/m^2^ in a multivariable model (p = 0.004). The greatest odds of EBL ≥ 1000 mL was observed among patients with BMI 50–59.9 kg/m^2^ (aOR 1.69 [1.28–2.21]), relative to patients with BMI 18.5–24.9 kg/m^2^ (Table 3). Additional variables that were associated with an increased odds of PPH included age greater than 35 years (aOR 1.12 [1.02–1.23]), history of PPH (aOR 1.80 [1.43–2.27]), and general anesthesia (aOR 1.87 [1.53–2.28]). Delivering on the West Coast was associated with significantly lower odds of PPH (aOR 0.62 [0.57–0.68]) (Table 3).

**Table 2:** Association between BMI and EBL at time of CD.

Pregnancy outcomes	All (n = 27,708)	BMI 18.5–24.9 (n = 2101)	BMI 25–29.9 (n = 7407)	BMI 30–34.9 (n = 8406)	BMI 35–39.9 (n = 5351)	BMI > 40 (n = 4443)	*p* value
Estimated blood loss							
< 1000 mL	22,497 (81.2%)	1738 (82.7%)	6006 (81.1%)	6927 (82.4%)	4269 (79.8%)	3557 (80.1%)	** < 0.001**
≥ 1000 mL	3293 (11.9%)	202 (9.6%)	878 (11.9%)	949 (11.3%)	697 (13.0%)	567 (12.8%)	
Missing/Unknown	1918 (6.9%)	161 (7.7%)	523 (7.1%)	530 (6.3%)	385 (7.2%)	319 (7.2%)	

*BMI* body mass index, *EBL* estimated blood loss.

Significant values are in bold.

**Table 3:** Model-based sensitivity analysis for the association between discretized BMI and EBL ≥ 1000 mL.

Logistic regression—risk factors for EBL ≥ 1000 mL				
Potential risk factors	Simple logistic regression		Multiple logistic regression^A^	
	Odds ratio	95% CI	Adjusted odds ratio^B^	95% CI
BMI				
18.5–24.9	1	Reference	1	Reference
25–29.9	1.21	1.03–1.43	1.22	1.03–1.43
30–39.9	1.23	1.05–1.43	1.25	1.06–1.46
40–49.9	1.26	1.06–1.50	1.29	1.07–1.54
50–59.9	1.69	1.29–2.21	1.69	1.28–2.21
> 60	1.86	0.10–3.46	1.85	0.98–3.47
Age (y)				
< 20	0.86	0.71–1.03	0.85	0.70–1.03
20–34	1	Reference	1	Reference
> 35	1.15	1.04–1.27	1.12	1.02–1.23
Race/Ethnicity				
Black	1	0.89–1.12	NIFM^D^	
White	1	Reference		
Asian	0.96	0.78–1.18		
Other/Unknown	0.86	0.79–0.94		
Marital status				
Single	0.88	0.82–0.95	NIFM^D^	
Married	1	Reference		
Other/Unknown	0.95	0.81–1.11		
Geographical area				
East coast	1.24	1.12–1.37	0.94	0.85–1.05
Central	1	Reference	1	Reference
West coast	0.96	0.89–1.04	0.62	0.57–0.68
Insurance				
Medicaid	0.71	0.65–0.77	0.65	0.59–0.72
Private	1	Reference	1	Reference
Uninsured	0.53	0.42–0.67	0.51	0.40–0.65
Other/Unknown	0.73	0.67–0.80	0.72	0.66–0.80
Parity				
0	1	Reference	NIFM^C^	
1	1.03	0.93–1.14		
2+	1.06	0.95–1.18		
Missing/Unknown	1.25	0.98–1.59		
Prior CD	0.94	0.86–1.03	NIFM^D^	
Asthma	0.88	0.65–1.21	NIFM^C^	
Pre-gestational	1.11	0.87–1.42	NIFM^C^	
Diabetes				
Chronic	1.16	0.93–1.45	NIFM^D^	
Hypertension				
Renal disease	1.36	0.28–6.59	NIFM^D^	
Smoking during	1.04	0.94–1.15	NIFM^C^	
Pregnancy				
History of PPH	1.96	1.57–2.45	1.8	1.43–2.27
EGA < 37 Weeks	1.09	0.97–1.22	NIFM^D^	
Placenta Previa	1.96	1.52–2.53	1.68	1.28–2.21
Intrapartum abruption	2.45	1.88–3.20	2.3	1.74–3.05
Placenta accreta	10.34	5.58–19.15	7.23	3.77–13.87
Preeclampsia	1.08	0.90–1.29	NIFM^C^	
Eclampsia	0.94	0.52–1.67	NIFM^C^	
Gestational diabetes	1.12	0.99–1.26	NIFM^D^	
HELLP	0.29	0.04–2.17	NIFM^D^	
Chorioamnionitis	1.19	0.83–1.72	NIFM^C^	
Venous thromboembolism	1.28	0.71–2.32	NIFM^C^	
Platelets < 150,000/mL	1.13	1.01–1.27	1.11	0.99–1.25
Hematocrit < 32%	1.08	0.99–1.18	1.1	1.00–1.20
General anesthesia	2.13	1.76–2.58	1.87	1.53–2.28
Magnesium sulfate use	1.2	1.05–1.38	NIFM^D^	
Oxytocin use	1.37	1.13–1.67	1.43	1.17–1.76
Dinoprostone use	1.13	0.99–1.29	NIFM^D^	
Misoprostol use	1.68	1.50–1.88	1.57	1.40–1.77
Any antibiotic use	2.43	2.08–2.83	2.68	2.28–3.14

*Y* year, *BMI* body mass index, *CD* cesarean delivery, *PPH* postpartum hemorrhage, *EGA* estimated gestational age, *HELLP* hemolysis, elevated liver enzymes and low platelets syndrome.

^A^Summary results from final model following iterative exclusion process.

^B^Employed purposeful selection methodology to adjust for potential confounds (prescreen for inclusion with Wald Test at α = 0.25 level, iterative exclusion at α = 0.10). See Bursac, Gauss, Williams, and Hosmer (2008) and Hosmer & Lemeshow (2000;1999).

^C^Excluded during prescreening at the α = .25 level.

^D^Excluded during the iterative process at the α = .10 level.

We found significant associations between BMI category and the frequency of secondary maternal outcomes in our cohort including pre-eclampsia (p < 0.001), gestational diabetes (*p* < 0.001), and any antibiotic use (*p* = 0.001) (Table 4). Considering secondary neonatal outcomes, BMI category was significantly associated with preterm delivery (*p* < 0.001) and NICU admission (*p* < 0.001) (Table 5). Categorical obesity was associated with increased odds of pre-eclampsia (aOR 1.92 [1.63–2.26]). Obesity was associated with decreased odds of preterm delivery at less than 37 weeks gestation (aOR 0.74 [0.69–0.80]), HELLP (aOR 0.30 [0.13–0.69]) and intrapartum placental abruption (aOR 0.67 [0.53–0.84]). Among patients with obesity, we observed an increased odds of oxytocin (aOR 1.26 [1.14–1.39]), dinoprostone (aOR 1.52 [1.37–1.68]), misoprostol (aOR 1.44 [1.30–1.59]), and magnesium sulfate usage (aOR 1.14 [1.02–1.26]). We observed a decreased odds of blood transfusion among patients with obesity (aOR 0.73 [0.55–0.97]) (Table 6).

**Table 4:** Secondary maternal outcomes of study cohort.

Maternal outcomes	All (n = 27,708)	BMI 18.5–24.9 (n = 2101)	BMI 25–29.9 (n = 7407)	BMI 30–34.9 (n = 8406)	BMI 35–39.9 (n = 5351)	BMI > 40 (n = 4443)	*p* value
Placenta previa	379 (1.4%)	51 (2.4%)	136 (1.8%)	103 (1.2%)	55 (1.0%)	34 (0.8%)	** < 0.001**
Intrapartum abruption	311 (1.1%)	49 (2.3%)	89 (1.2%)	100 (1.2%)	35 (0.7%)	38 (0.9%)	** < 0.001**
Placenta accreta	49 (0.2%)	2 (0.1%)	17 (0.2%)	16 (0.2%)	9 (0.2%)	5 (0.1%)	0.607
Intrapartum bleeding	125 (0.5%)	13 (0.6%)	33 (0.5%)	39 (0.5%)	23 (0.4%)	17 (0.4%)	0.760
Pre-eclampsia	1153 (4.2%)	41 (2.0%)	167 (2.3%)	294 (3.5%)	293 (5.5%)	358 (8.1%)	** < 0.001**
Eclampsia	111 (0.4%)	12 (0.6%)	16 (0.2%)	28 (0.3%)	31 (0.6%)	24 (0.5%)	**0.004**
Gestational diabetes	2406 (8.7%)	72 (3.4%)	424 (5.7%)	649 (7.7%)	586 (11.0%)	675 (15.2%)	** < 0.001**
HELLP	23 (0.08%)	4 (0.2%)	10 (0.1%)	5 (0.06%)	4 (0.07%)	0 (0.0%)	**0.027**
Chorioamnionitis	246 (0.9%)	21 (1.0%)	73 (1.0%)	90 (1.1%)	39 (0.7%)	23 (0.5%)	**0.013**
Venous thromboembolism	92 (0.3%)	8 (0.4%)	19 (0.3%)	22 (0.3%)	15 (0.3%)	28 (0.6%)	**0.005**
ICU Admission	44 (0.2%)	7 (0.3%)	13 (0.2%)	10 (0.1%)	5 (0.1%)	9 (0.2%)	0.143
Blood transfusion	199 (0.7%)	19 (0.9%)	62 (0.8%)	55 (0.7%)	29 (0.5%)	34 (0.8%)	0.245
Platelets < 150,000/μL	2876 (10.4%)	285 (13.6%)	985 (13.3%)	901 (10.7%)	468 (8.8%)	237 (5.3%)	** < 0.001**
Hematocrit < 32%	5090 (18.4%)	493 (23.5%)	1456 (19.7%)	1575 (18.7%)	889 (16.6%)	677 (15.2%)	** < 0.001**
General anesthesia	622 (2.2%)	80 (3.8%)	182 (2.5%)	163 (1.9%)	106 (2.0%)	91 (2.1%)	** < 0.001**
Magnesium sulfate use	1767 (6.4%)	151 (7.2%)	392 (5.3%)	492 (5.9%)	363 (6.8%)	369 (8.3%)	** < 0.001**
Oxytocin use	25,722 (92.8%)	1905 (90.7%)	6812 (92.0%)	7829 (93.1%)	5003 (93.5%)	4173 (93.9%)	** < 0.001**
Dinoprostone use	2138 (7.7%)	104 (5.0%)	491 (6.6%)	664 (7.9%)	431 (8.1%)	448 (10.1%)	** < 0.001**
Misoprostol use	2175 (7.9%)	108 (5.1%)	497 (6.7%)	658 (7.8%)	467 (8.7%)	445 (10.0%)	** < 0.001**
Any antibiotic use	23,796 (85.9%)	1813 (86.3%)	6456 (87.2%)	7210 (85.8%)	4540 (84.8%)	3777 (85.0%)	**0.001**

*HELLP* hemolysis, elevated liver enzymes and low platelets syndrome, *ICU* intensive care unit.

Significant values are in bold.

**Table 5:** Secondary neonatal outcomes of study cohort.

Neonatal outcomes	All (n = 27,708)	BMI 18.5–24.9 (n = 2101)	BMI 25–29.9 (n = 7407)	BMI 30–34.9 (n = 8406)	BMI 35–39.9 (n = 5351)	BMI > 40 (n = 4443)	*p* value
Estimated gestational age							
< 37 weeks	3093 (11.2%)	368 (17.5%)	835 (11.3%)	854 (10.2%)	536 (10.0%)	500 (11.3%)	** < 0.001**
37 + weeks	24,164 (87.2%)	1693 (80.6%)	6454 (87.1%)	7418 (88.3%)	4732 (88.4%)	3867 (87.0%)	
Missing/Unknown	451 (1.6%)	40 (1.9%)	118 (1.6%)	134 (1.6%)	83 (1.6%)	76 (1.7%)	
NICU Admission	1714 (6.2%)	160 (7.6%)	420 (5.7%)	472 (5.6%)	325 (6.1%)	337 (7.6%)	** < 0.001**

*NICU* neonatal intensive care unit.

Significant values are in bold.

**Table 6:** Model-based OR/aOR for the effect of dichotomized obesity on obstetric outcomes.

Logistic regression—obesity (BMI > 30) as a predictor of pregnancy outcomes				
Pregnancy outcomes	Simple logistic regression		Multiple logistic regression	
	Odds ratio	95% CI	Adjusted Odds ratio^A^	95% CI
EBL ≥ 1000 mL	1.07	0.99–1.16	1.07	0.99–1.16
Placenta previa	0.53	0.43–0.65	0.52	0.43–0.64
Intrapartum abruption	0.65	0.52–0.82	0.67	0.53–0.84
Placenta accreta	0.83	0.46–1.47	–	–
Intrapartum bleeding	0.90	0.62–1.29	–	–
Preeclampsia	2.45	2.10–2.85	1.92	1.63–2.26
Eclampsia	1.55	1.01–2.38	1.53	0.99–2.35
Gestational Diabetes	2.13	1.92–2.36	2.18	1.96–2.42
HELLP	0.34	0.15–0.78	0.30	0.13–0.69
Chorioamnionitis	0.84	0.65–1.09	0.90	0.69–1.17
Venous thromboembolism	1.26	0.80–1.97	–	–
EGA < 37 Weeks	0.80	0.74–0.87	0.74	0.69–0.80
ICU admission	0.63	0.35–1.13	–	–
NICU admission	1.02	0.92–1.13	–	–
Perinatal blood transfusion	0.76	0.57–1.01	0.73	0.55–0.97
Platelets < 150,000/μL	0.63	0.58–0.68	0.62	0.58–0.67
Hematocrit < 32%	0.81	0.76–0.86	0.75	0.70–0.80
General anesthesia	0.71	0.61–0.84	0.70	0.59–0.82
Magnesium sulfate use	1.19	1.07–1.32	1.14	1.02–1.26
Oxytocin use	1.29	1.18–1.42	1.26	1.14–1.39
Dinoprostone use	1.39	1.26–1.53	1.52	1.37–1.68
Misoprostol use	1.39	1.26–1.53	1.44	1.30–1.59
Any antibiotic use	0.87	0.81–0.94	0.96	0.88–1.04

*EBL* estimated blood loss, *HELLP* hemolysis, elevated liver enzymes and low platelets syndrome, *EGA* estimated gestational age, *ICU* intensive care unit, *NICU* neonatal intensive care unit.

^A^Adjusted for confounding maternal characteristics employing a purposeful selection methodology (prescreen for inclusion with Wald Test at α = 0.25 level, iterative exclusion at α = 0.10). See Bursac, Gauss, Williams, and Hosmer (2008) and Hosmer & Lemeshow (2000;1999).

## Discussion

In our cohort of 27,708 CD across the United States, we observed an association between increasing BMI and EBL ≥ 1000 mL after adjusting for maternal demographic characteristics known to be associated with PPH. The observation that further discretized BMI was associated with increased odds of EBL ≥ 1000 mL while categorical obesity was not suggests that the BMI cutoff of ≥ 30 kg/m^2^ BMI to define obese versus non-obese is not itself predictive of PPH. We found an association between obesity and other intrapartum complications, including pre-eclampsia and gestational diabetes. Despite an increased odds of EBL ≥ 1000 mL, we found a decreased odds of blood transfusion in patients with obesity after CD.

Our findings contrast with prior work investigating the relationship between obesity and PPH. Butwick et al. conducted a large population-based cohort study that found an increased odds of hemorrhage after vaginal delivery and decreased odds of hemorrhage after CD for women with obesity; however, the effect was small and without strong evidence of a dose–response relationship between BMI and PPH^9^. In addition, the absolute event rate for PPH was 2.8%, which was lower than the frequency observed in our cohort (11.9%)^9^. Paglia et al. found a reduced risk of severe PPH among women with obesity after controlling for mode of delivery; of note, this was a case–control study of women with PPH who received blood components, indicating severe hemorrhage^11^. Our study focused on EBL ≥ 1000 mL, which may or may not have clinical effects.

Interestingly, despite an increased odds of PPH, we observed a decreased odds of blood transfusion among women with obesity. This finding implies that the increased blood loss we observed in patients with obesity may lack clinical relevance, and is consistent with prior work demonstrating greater blood volume and hemoglobin mass in individuals with obesity^25^. This finding also lends support to the notion that estimated blood loss is an unreliable indicator of clinically significant hemorrhage, and variation exists among hospitals in the use of quantitative over estimated blood loss. One formula devised for estimating allowable blood loss prior to transfusion includes allowable blood loss as directly proportional to the patient’s estimated blood volume, implying that patients with obesity with a higher estimated blood volume are able to tolerate a higher estimated blood loss prior to requiring blood transfusion^26^. However, there is little consensus in the literature regarding the impact of obesity on maternal blood volume, with one study reporting similar circulating blood volume between gravidas with and without obesity^27^.

We found that women with obesity in our cohort more frequently used oxytocin, dinoprostone, misoprostol, and magnesium sulfate, lending support to the theory that the mechanism by which obesity could increase PPH risk is related to uterine atony. Future work may examine variation in the etiology of PPH among women with and without obesity, as well as variations in PPH management based on BMI. The impact of obesity on maternal blood volume and the threshold for estimated blood loss at which clinically significant changes in hematologic parameters or transfusion risk occurs in women with obesity should also be investigated further due to our finding that despite a higher estimated blood loss, transfusion was not more frequent among women with obesity.

Limitations of our study include limitations common to studies using large databases, including potential misclassification errors and missing data. To address this limitation, we treated missing data as missing at random and employed multiple imputation by chained equations to account for statistical uncertainty while addressing missing data. Our dataset was unable to distinguish quantitative blood loss from estimated blood loss, which limits the accuracy of the blood loss reported and may contribute to variation in reporting blood loss among the hospitals in our dataset. Pre-pregnancy BMI and gestational weight gain were not reliably captured in our dataset, yet may affect pregnancy outcome more significantly than BMI at the time of delivery. Our study is also limited by the lack of ability to capture postpartum complications that occur after hospital discharge, such as delayed PPH, which is a potential explanation for the lack of association we observed between obesity and venous thromboembolism in our study despite the known increased risk of venous thromboembolism among gravidas with obesity^6,28^.

Despite these limitations, our study has a number of strengths. We included a large number of patients across multiple sites in different geographic regions across the United States, increasing the generalizability of our results. We excluded patients with missing BMI data since this variable was the focus of our study. We focused on including only CD to eliminate the potential confounding effect of mode of delivery in the relationship between BMI and blood loss. Our findings have significant clinical implications due to the rising prevalence of obesity in the United States as well as the high number of CD performed annually, currently accounting for more than 30 percent of all deliveries in the United States^29^. Our results demonstrate several potential risk factors for PPH after CD that can be used for population-based risk assessment, which can aid clinicians in identifying groups of patients that are at highest risk for PPH as part of an ongoing effort to lower hemorrhage-related morbidity and mortality.

In conclusion, we found an association between BMI and estimated blood loss after CD, with elevated BMI associated with an increased frequency of PPH. Despite a higher estimated blood loss, we did not find a higher frequency of blood transfusion among women with obesity after CD, implying that there may be no difference in clinically significant blood loss between women with and without obesity (Supplementary Information). Future work may investigate the amount of blood loss at delivery associated with maternal morbidity among patients with obesity, as well as variation in the etiology and management of PPH based on BMI.

## Supplementary Information

Supplementary Information.

## Data Availability

The dataset analyzed during the current study is not publicly available due to the sensitive nature of de-identified electronic health record data. To request more information about the data analyzed for this study, the principal investigator (Dr. Homa Ahmadzia) or the corresponding author (Dr. Julia Whitley) may be contacted.
