# Supplementary material for: The association between body mass index and postpartum hemorrhage after cesarean delivery
Source: Sci Rep. 2023 Jul 25;13:11998. doi: 10.1038/s41598-023-38526-7 (PMC10368616; doi:10.1038/s41598-023-38526-7)
Supplement: Supplementary file 1 — Supplementary Information. [file 41598_2023_38526_MOESM1_ESM.docx]

In Table 6, we report model-based odds ratios and adjusted odds ratios for the effect of dichotomized obesity (BMI ≥30 kg/m^2^) on obstetric outcomes. The effect of obesity on each outcome was examined separately as follows:

For the effect of obesity on EBL ≥1000 mL, covariates included BMI, age, insurance, geographical area, and prior cesarean delivery. For the effect of obesity on placenta previa, covariates included BMI, age, race, geographical area, and parity. For the effect of obesity on intrapartum abruption, covariates included BMI, race, geographical area, and parity. For the effect of obesity on pre-eclampsia, covariates included BMI, tobacco use, insurance, race, geographical area, parity, pre-gestational diabetes, and hypertension. For the effect of obesity on eclampsia, covariates included BMI, age, geographical area, and parity. For the effect of obesity on gestational diabetes, covariates included BMI, age, insurance, race, geographical area, parity, prior cesarean delivery, asthma, pre-gestational diabetes, and hypertension. For the effect of obesity on HELLP, covariates included BMI, geographical area, and hypertension. For the effect of obesity on chorioamnionitis, covariates included BMI, age, insurance, race, prior cesarean delivery, and hypertension. For the effect of obesity on preterm delivery, covariates included BMI, age, insurance, race, marital status, geographical area, parity, asthma, pre-gestational diabetes, and hypertension. For the effect of obesity on blood transfusion, covariates included BMI, insurance, race, geographical area, and parity. For the effect of obesity on thrombocytopenia, covariates included BMI, tobacco use, age, race, geographical area, parity, and pre-gestational diabetes. For the effect of obesity on hematocrit less than 32 percent, covariates included BMI, tobacco use, age, insurance, race, geographical area, parity, prior cesarean delivery, and pre-gestational diabetes. For the effect of obesity on general anesthesia, covariates included BMI, tobacco use, insurance, race, geographical area, prior cesarean delivery, and pre-gestational diabetes. For the effect of obesity on magnesium sulfate use, covariates included BMI, age, insurance, race, geographical area, parity, prior cesarean delivery, pre-gestational diabetes, and hypertension. For the effect of obesity on oxytocin use, covariates included BMI, tobacco use, age, insurance, race, geographical area, and prior cesarean delivery. For the effect of obesity on dinoprostone use, covariates included BMI, age, insurance, race, geographical area, parity, prior cesarean delivery, and hypertension. For the effect of obesity on misoprostol use, covariates included BMI, tobacco use, age, insurance, race, geographical area, parity, prior cesarean delivery, asthma, and hypertension. For the effect of obesity on antibiotic use, covariates included BMI, tobacco use, insurance, race, geographical area, parity, prior cesarean delivery, pre-gestational diabetes, and hypertension.
